# A YOUNG LADY PRESENTED WITH LIMITED PULMONARY WEGENER'S GRANULOMATOSIS

**DOI:** 10.4103/0970-2113.45286

**Published:** 2008

**Authors:** Atin Dey, Datta Chaudhuri Arunabha, Pandit Sudipta, Kundu Susmita, Saha Mita

**Affiliations:** Dr. Atin Dey, Hemangini Nagar, Block-I, Flat No.–202, 53, New G.T.Road, P.O. Uttarpara, District –Hooghly, Pin – 712258. E-mail: dratindey@yahoo.co.uk

**Keywords:** Limited Pulmonary Wegener's Granulomatosis, C-ANCA

## Abstract

A 19 year old female college student presented with fever, dry cough, chest pain, blood tinged sputum with subsequent development of polyarthralgia with radiological evidence of bilateral multiple unevenly distributed pulmonary nodular opacities with cavitation. There was no other systemic involvement and the patient was cytoplasmic antineutrophil antibody (c-ANCA) positive with more than four times the normal upper limit of anti PR3 antibody. Excellent response to oral steroid with antimicrobial agent “trimethoprim – sulphamethoxazole” was noted.

## INTRODUCTION

Wegener's granulomatosis is a unique clinico-pathological disease entity characterized by necrotizing granulomatous vasculitis of the upper and lower respiratory tract, focal necrotizing glomerulonephritis, generalized focal necrotizing vasculitis involving both arteries and veins^1^ . In practical terms the demonstration of one or more of these in combination with positive serological evidence is now generally accepted as fulfilling the criteria^2^. Although in its classical form Wegener's granulomatosis is a multisystem disease, there can actually be a spectrum of clinical manifestation and the disease may present with limited organ involvement^3^. In so called limited Wegener's granulomatosis, there is no kidney disease and no evidence of systemic vasculitis^1^. Where the clinical findings are typical or consistent and there is a positive c-ANCA, tissue may not be necessary to establish the diagnosis^1^. There are no absolutely diagnostic pathological features of Wegener's granulomatosis^1^. A transbronchial lung biopsy may occasionally support diagnosis, but more tissue from thoroscopic or open lung biopsy may be required if evidence elsewhere is not supportive^1^.

## CASE REPORT

A 19 year old unmarried female college student was admitted in our hospital with history of right sided chest pain, dry cough, evening rise of temperature and occasional blood tinged sputum since one month. There was no history of breathlessness, joint pain or urinary complaint. Her appetite was decreased and she was losing weight. The disease started insidiously with right sided chest pain with subsequent development of dry cough followed by blood tinged sputum. Subsequently few days after her admission she developed arthralgia of multiple large and small joints.

She did not have any concomitant general illness or any symptoms referable to any other system. No family history of a similar or related illness was reported. Her menstrual history was normal.

On examination, the patient was of average built. There was moderate pallor but jaundice, odema, cyanosis and clubbing were absent. Pulse rate was 86/minute regular, blood pressure – 110/70 mm of hg, respiratory rate 18/minute. Chest was clear on percussion and auscultation. Examination of cardiovascular system, central nervous system and gastro-intestinal system were normal. Detailed ear, nose, throat and eye examinations were normal. There were no suspicious lesion in nose, pharynx, oral cavity.

Routine laboratory examinations revealed haemoglobin 8gm/dl, ESR 40mm/first hour, total leucocyte count 9500 of which neutrophil is 80% and normal platelet count. Serum glucose level, renal and liver function tests were normal. Sputum examination was negative for Acid Fast Bacillus, malignant cell and fungal hyphae. Repeated urinanalysis for sediment, cast, proteinuria, RBC was normal. Ultrasonography (USG) of whole abdomen revealed no abnormality. Patient was tuberculin negative. Chest radiography showed bilateral multiple unevenly distributed nodular opacities, with few of them showing cavitation, sparing both the apices with coalescence of some nodules (Fig. I). X-ray and computerised tomography (CT) scan of para nasal sinuses were within normal limits. CT scan of thorax revealed multiple nodular lesions of varying sized from one to few centimeters in both lungs, some of them were parenchymal, some were subpleural in location sparing the apices. Few nodules showed central necrosis with cavitation. There was no mediastinal lymphadenopathy, no pleural effusion (Fig. II). Fine needle aspiration cytology (FNAC) obtained from one of the parenchymal nodules showed non specific inflammation with no malignant cells or granuloma . Fibre optic bronchoscopy (FOB) revealed no naso-tracheal or endobronchial lesion, mucosal alteration or any bleeding points. Analysis of broncho alveolar lavage (BAL) fluid revealed mononuclear cell preponderance. Smear, culture and Papanicolaou (PAP) stain of BAL fluid were negative for significant pathogens including Mycobacterium tuberculosis, fungus or malignant cells.

**Fig 1:**
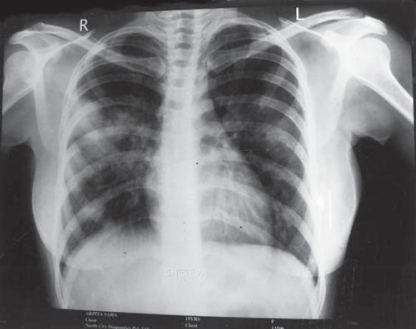
Initial chest radiograph showing bilateral pulmonary unevenly distributed nodular opacities with coalescence of some pulmonary nodules.

**Fig 2:**
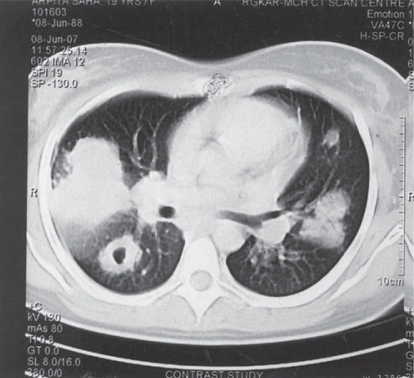
CT scan of thorax (mediastinal view) showing bilateral multiple pulmonary nodules of varying size with cavitation in one nodule with no mediastinal lymphadenopathy.

Estimation of serum calcium and angiotensin converting enzyme were within normal limits. Serum examination for rheumatoid factor and anti nuclear factor (ANF) were negative. Pulmonary function test was normal.

The serum perinuclear antineutrophil cytoplasmic antibody (p-ANCA) was negative but serum cytoplasmic antineutrophil antibody (c-ANCA) was positive (20.66 U/ml) with more than four times the normal upper limit (reference range < 5 U/ml ) of anti PR3 antibody done by enzyme linked immuno sorbant assay (ELISA) method.

## TREATMENT

She had been offered treatment with cyclophosphamide and steroid but she and her parents were not willing to take cyclophosphamide because of its possible adverse effects. She was put on a daily combination regimen of prednisolone 1mg/kg and “sulphamethaxazole-trimethoprim” combination (cotrimoxazole-DS twice daily) orally. One month after the ongoing treatment the lung nodules had regressed in size while few of them completely disappeared with a dramatic improvement of her symptoms. Follow up chest x-ray after two months of ongoing treatment showed disappearance of nearly all nodular lesions (Fig. III ). Serum c-ANCA level was also reduced to normal.

**Fig 3:**
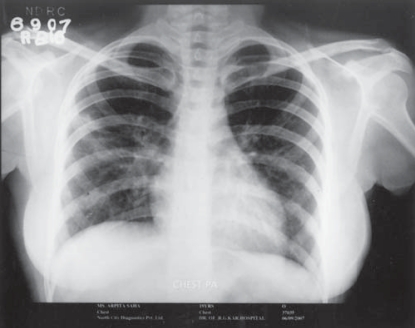
Chest radio graph showing disappearance of nodules after treatment.

## DISCUSSION

In 1966, Carrington and Liebow^4^ described 16 patients with pulmonary lesion identical to those of Wegener's Granulomatosis with absence of or limited lesion elsewhere and particularly no focal glomerulitis. They described it as “Limited form of Angitis and Granulomatisis of Wegener's type”. Cassan and Associate^5^ supported the concept of limited forms of Wegener's Granulomatosis by reporting four cases in 1970. Its clinical picture is rather nonspecific and diversified, ranging from no symptoms to cough, dyspnoea, haemoptysis, pleuritic chest pain accompanied by malaise, weight loss or fever^4^,^6^,^7^ Sometimes a lesion may be discovered at routine radiographic examination of an asymptomatic patient^2^. There may be involvement of other tissues such as the skin and eye^6^,^8^,^9^ Chest X-ray in this condition shows pulmonary lesions predominantly in the lower lobes and the most common form is a discrete lesion greater than 1cm. in diameter and in two thirds of cases the lesions are multiple and bilateral ; and in one third cases cavitation occurs.^2^ CECT Scan of the thorax can show nodular and cavitating lesions which are not obvious on chest radiography.^11^ Pulmonary lesions in Limited Wegener`s.Granulomatosis are similar to that of Classical form.^4^,^16^

Antineutrophilic cytoplasmic antibodies (ANCA) have a high degree of association with Wegener's. Granulomatosis. Two main immunofluroescent patterns of ANCA have been described; a Cytoplasmic pattern c-ANCA and a perinuclear pattern p-ANCA, positivity of both have broadly different association. The contribution of ANCA to the pathogenesis of Wegener's.Granulomatosis is unclear, but they appear to be responsible for some portion of the immune response that leads to disease^10^. The c-ANCA is positive in more than 90% of patients with active Wegener's Granulomatosis^11^,^12^ In Limited Wegener`s Granulomatosis, that is without active glomerulonephritis, sensitivity of c-ANCA is 65-70 percent^12^ . None of the studies of c-ANCA in normal individuals showed positive result^11^,^13^. The c-ANCA associated with Wegener's Granulomatosis is almost always an anti PR3(Proteinase 3) antibody. Therefore, it is prudent to confirm a finding of c-ANCA by specific Elisa assay for Anti PR3 or Anti MPO (Myeloperoxidase)^10^. A four fold rise of c-ANCA titre even in the absence of renal involvement is quite specific for diagnosis of Wegener's Granulomatosis^14^. Response to the treatment is another evidence in favour of diagnosis of Wegener's Granulomatosis.

Malignancy, infection, connective tissue disorders, granulomatous diseases are the differential diagnosis of Limited Pulmonary Wegener's Granulomatosis. Early diagnosis and prompt treatment is crucial as the disease is a life threatening disorder and the drugs required to treat Wegener's Granulomatosis are potentially toxic, therefore a definitive diagnosis is essential.^14^ The definitive diagnosis of Wegener's Granulomatosis is made by tissue biopsy. Where the clinical findings are typical or consistent, with positive c-ANCA, tissue biopsy may not be necessary to establish the diagnosis^1^. There are no absolute diagnostic pathologic features of Wegener's Granulomatosis.^1^

The treatment of choice for Wegener's Granulomatosis is Cyclophophamide and Prednisolone^1^ These result in remission of the disease in more than 90% of patients^15^. In patients who do not tolerate Cyclophophamide , other cytotoxic drugs like Azathioprine have been used. The antimicrobial agent “Trimethoprim Sulfamethoxazole” may be useful in therapy of Limited Wegener's Granulomatosis or as an adjunct to therapy^1^. De Remee and Colleagues reported in 1985 that “Trimethoprim Sulfamethoxazole” combination brought improvement in 11 out of 12 patients with Wegener's Granulomatosis^16^. The action of “Trimethoprim Sulfamethoxazole” in Wegener's Granulomatosis is anti inflammatory through interference with the formation of oxygen derived radicals by activated neutrophils as suggested by some research worker, although other workers assert that action of “Trimethoprim Sulfamethoxazole” in Wegener's Granulomatosis are related to its antimicrobial effect^17^.

Our patient presented with chest pain, dry cough, fever, haemoptysis, loss of appetite and showed multiple bilateral nodular opacities on chest radiograph. These are the usual presentation of some common disorders like secondary metastases of lung, pulmonary tuberculosis, sarcoidosis ,fungal infection of lung, rheumatoid nodule etc. Hence thorough clinical examination and all possible relevant investigation were done to exclude those common disorders. Four fold rise of c-ANCA, presence of Anti PR3 antibody even in the absence of renal involvement is quite specific for diagnosis of Wegener's Granulomatosis. The study of this case emphasizes that Wegener Granulomatosis must be considered when assessing patients presenting with common respiratory symptoms with multiple nodular opacities on chest radiograph, in the absence of primary or secondary malignancy, tuberculosis, fungal disease, sarcoidosis and rheumatoid nodule. Where Cyclophosphamide can't be given an alternative to it may be oral “Trimethoprim Sulfamethoxazole” along with Prednisolone . Since non renal Wegener`s Granulomatosis may change the pattern of disease to involve kidney , long term follow up is essential.^18^
